# The cerebro-pelvic axis: a unified framework linking higher brain function, pelvic floor control, and lower urinary tract dysfunction

**DOI:** 10.3389/fnins.2026.1773086

**Published:** 2026-03-23

**Authors:** Zhong Li, Jing Fu, Jianlin Pu, Ying Qian, Xuesong Gai, Li Li

**Affiliations:** 1 Second Clinical Medical College, Yunnan University of Chinese Medicine, Kunming, China; 2 School of Traditional Dai Medicine, West Yunnan University of Applied Sciences, Xishuangbanna, China; 3 School of Medicine, Kunming University of Science and Technology, Kunming, China; 4 Department of Rehabilitation Medicine, The First People's Hospital of Yunnan Province (The Affiliated Hospital of Kunming University of Science and Technology), Kunming, China; 5 Department of Emergency Trauma Surgery, The First People's Hospital of Yunnan Province (The Affiliated Hospital of Kunming University of Science and Technology), Kunming, China

**Keywords:** Alzheimer's disease, cerebro-pelvic axis, disorders of consciousness, interoception, neuromodulation, neuro-pelviology, urinary incontinence

## Abstract

Clinical practice often treats higher brain disorders (e.g., Alzheimer's disease and prolonged disorders of consciousness) and pelvic floor dysfunction (e.g., stress urinary incontinence and overactive bladder) as unrelated problems, despite frequent co-occurrence and overlapping vulnerability contexts (e.g., aging, frailty, medications). Here, “axis” denotes a control-architecture mapping and phenotyping heuristic for LUT control and pelvic-floor outlet coordination, rather than a claim of new anatomy or shared etiology. Accordingly, we use a hypothesis-generating control-loop framing that links descending executive control with ascending interoceptive signaling to account for this clinicobiological mismatch. Within this framework, two provisional working failure-mode categories: top-down disintegration, in which impaired supraspinal control weakens volitional inhibition and shifts continence toward reflex-dominant regulation; and bottom-up disturbance, in which persistent peripheral salience-like signals may up-weight interoceptive processing and contribute to maladaptive central network adaptations. These categories are LUT-focused working categories and are not intended as a comprehensive taxonomy of all LUT phenotypes. We further introduce Coordinated Axis Neuromodulation (CAN) as a hypothesis-driven intervention concept that temporally couples cortical, spinal, and peripheral stimulation and may facilitate control-loop–level rebalancing compared with single-node modulation; this proposal requires direct empirical validation. This framework yields testable predictions, including directionally specific coupling between cortical biomarkers (e.g., executive/salience network metrics) and peripheral readouts (e.g., pelvic-floor EMG timing indices and/or diary-defined urgency/UUI burden; urodynamics as supportive phenotyping/secondary mechanistic data when included), and differential response profiles of CAN protocols across failure-mode–stratified cohorts. We outline a validation route spanning synchronized neurophysiology–pelvic physiology paradigms (e.g., EMG timing and diary endpoints; urodynamics as supportive phenotyping/secondary mechanistic data when included), proof-of-mechanism studies, and safety-monitored, mechanism-oriented RCTs designed to falsify or refine the CPA/CAN hypothesis.

## 1 Introduction

Against the backdrop of a global aging population and the escalating burden of chronic diseases, an increasingly recognized clinical and conceptual disconnect: the recurring co-occurrence of higher-brain dysfunction and pelvic-floor/LUT symptoms in some populations remains artificially compartmentalized within conceptual frameworks and clinical practice (Bartolone et al., 2021). Taking Alzheimer's disease (AD) as an example, as cognitive and memory networks gradually deteriorate, urinary control often declines simultaneously (Bartolone et al., 2021). However, within the current healthcare system, these two processes are assigned to different specialties. AD is regarded as a classic neurodegenerative disorder, while the urinary incontinence associated with AD is often downgraded to a common geriatric issue or a late-stage caregiving burden. Rarely are these two conditions viewed as different manifestations that may be mapped onto a shared control architecture for coordinated phenotyping and management planning, rather than as evidence of shared pathogenesis (Gill et al., 2005; de Codt et al., 2015). A similar distinction exists in the management of patients with prolonged disorders of consciousness (pDoC). While the clinical focus for pDoC primarily centers on promoting arousal within the cortical-thalamic network, a patient's ability to truly be discharged from hospital care largely depends on whether they can be weaned off urinary catheters and regain basic bladder and/or bowel autonomy (Pelizzari et al., 2023). The restoration of consciousness and pelvic floor control, two functions that collectively determine a patient's dignity and prognosis, remain independently addressed in both research and treatment pathway. The core contradiction lies in the fact that the prevalence of this co-occurrence raises a testable question about whether partially overlapping control-circuit vulnerability patterns may be identifiable at the level of control-loop phenotyping, yet current disciplinary barriers hinder direct testing of this connection. Current neurodegeneration-focused and pelvic-floor–focused literatures often discuss cognitive decline in the brain and disruption of urinary control in discipline-specific terms, effectively treating them as parallel pathological trajectories in practice; this separation can also extend to conditions often framed as seemingly localized problems (Jafarian et al., 2025; Szabo et al., 2025). For example, stress urinary incontinence (SUI) is classically defined as an outlet-support/mechanical failure. Beyond this primary mechanism, some patients—particularly those with mixed urinary incontinence, coexisting urgency, or high symptom-related distress—may experience recurrent leakage episodes and anticipatory fear associated with increased symptom vigilance and psychosocial burden (Li and Wang, 2024). In this paper, these central effects are treated as subgroup-dependent aggravation mechanisms rather than core mechanistic evidence for pure SUI pathogenesis.

Whether the presenting disorder is higher-brain dominant (e.g., AD/pDoC) or pelvic-floor dominant (e.g., SUI), the CPA hypothesis maps these conditions onto different nodes of a shared brain–spinal–pelvic control architecture. In this framing, the “continuity” refers to the organization of the control loop (and the possibility of coupled dysfunction across nodes), not to a claim of shared etiology or direct disease-to-disease causality. Scientifically, we possess extensive knowledge relating to how brain networks collapse or how urethral pressure changes, yet we rarely couple these processes within an operationally testable integrative control-loop model. Clinically, patients receive fragmented and multi-targeted interventions combining cognitive medications, nursing pads, and arousal therapy that is disconnected from urinary management. This approach lacks layered, coordinated intervention strategies informed by a shared control-architecture perspective, representing an underdeveloped area for mechanism-oriented translational research.

To address this integrative gap, we draw conceptually on established “axis” models (e.g., the brain–gut axis) as an analogy and precedent for systems-level thinking, while recognizing that mechanistic mapping in the brain–gut research literature is more mature and that the CPA remains hypothesis-driven. This framework is used to describe the complex bidirectional communication between the brain and pelvic system through neural, endocrine, and immune pathways. This strategy has helped organize and interpret comorbid conditions such as anxiety disorders and irritable bowel syndrome, driven interdisciplinary research and fostering novel therapeutic approaches (Carabotti et al., 2015). Drawing upon this model, we have re-examined existing explorations in the pelvic-floor medicine literature. In a previous study, Professor Derek Griffiths demonstrated that urination is a complex decision-making process that is dependent on prefrontal inhibition and an emotional state, rather than a local reflex (Griffiths, 2015). Furthermore, the emerging clinical subspecialty of neuropelveology has enhanced our understanding of peripheral neuropathology in the pelvic floor, particularly in conditions of chronic pain (Cho, 2017). However, the former's research scope is typically confined to lower urinary tract control and is rarely integrated with the systemic collapse of higher cognitive networks. The latter approach primarily originated from the pelvic region and focuses on peripheral neuropathy and surgical reconstruction, without incorporating higher-level cognitive functions, emotions, and consciousness as equal components within its core framework. Consequently, an operationally useful integrative mapping framework for cross-level control-loop phenotyping across this spectrum remains underdeveloped.

Prior frameworks have described brain–bladder communication and supraspinal control circuits in substantial detail (e.g., the bladder–brain axis perspective). Our proposal does not redefine these canonical neurourological pathways. Instead, CPA is introduced as an explicit control-loop framing that: (i) broadens the mapping scope from “bladder” to the broader pelvic effector set (pelvic floor, outlet control, bowel/sexual functions when relevant); (ii) uses two directional working failure-mode categories (top-down disintegration vs. bottom-up disturbance) as a phenotyping language across neurologic and pelvic-floor–dominant conditions; and (iii) links this phenotyping to mechanism-oriented, falsifiable predictions and intervention design logic (CAN). Accordingly, CPA should be read as a hypothesis-generating synthesis layer built on established brain–bladder circuitry, rather than a replacement of existing neurourology models.

Importantly, CPA is proposed as a control-architecture and phenotyping framework, not a nosological claim. It does not assume a shared etiology or deterministic disease coupling between AD/pDoC and pelvic-floor disorders but instead provides a testable way to map how dysfunction at different nodes may produce partially coupled phenotypes in well-phenotyped cohorts.

In this Hypothesis and Theory article, we use the term “cerebro-pelvic axis” (CPA) as a hypothesis-generating, working control-loop mapping framework for organizing bidirectional brain-pelvic observations relevant to lower urinary tract control and pelvic-floor outlet coordination. Within this framework, interactions between higher brain functions (cognition, emotion, and consciousness) and pelvic organ/pelvic-floor functions (including urinary storage and voiding, and potentially bowel/sexual functions in broader applications) are organized as components of a brain-spinal-pelvic control loop. Based on this CPA framing, we outline an interdisciplinary research agenda (here termed “neuro-pelviology” as a provisional working label) for integrative, mechanism-oriented, and translational studies across brain-pelvis interactions. The core aim of the CPA framework is not to redefine disease entities, but to provide a control-architecture mapping for phenotyping how dysfunction may arise at different nodes of the brain–spinal–pelvic loop. Within this mapping, higher-brain–dominant and selected pelvic-floor–dominant presentations can be described as different failure-mode expressions, without implying shared etiology or disease-to-disease coupling. The present framework provisionally uses two working failure-mode categories, top-down disintegration (impaired descending control over storage–voiding coordination) and bottom-up disturbance (aberrant or over-weighted pelvic afferent/interoceptive signaling with potential central consequences in selected phenotypes). Here, “top-down disintegration” refers to impaired executive/brainstem command over storage–voiding coordination, whereas “bottom-up disturbance” refers to excessive or distorted bladder/pelvic afferent signaling that drives maladaptive central processing. This framework is intended to support integrated phenotyping and to generate testable hypotheses for diagnostic and intervention development.

## 2 CPA as a working control-loop mapping framework

**Figure 1:**
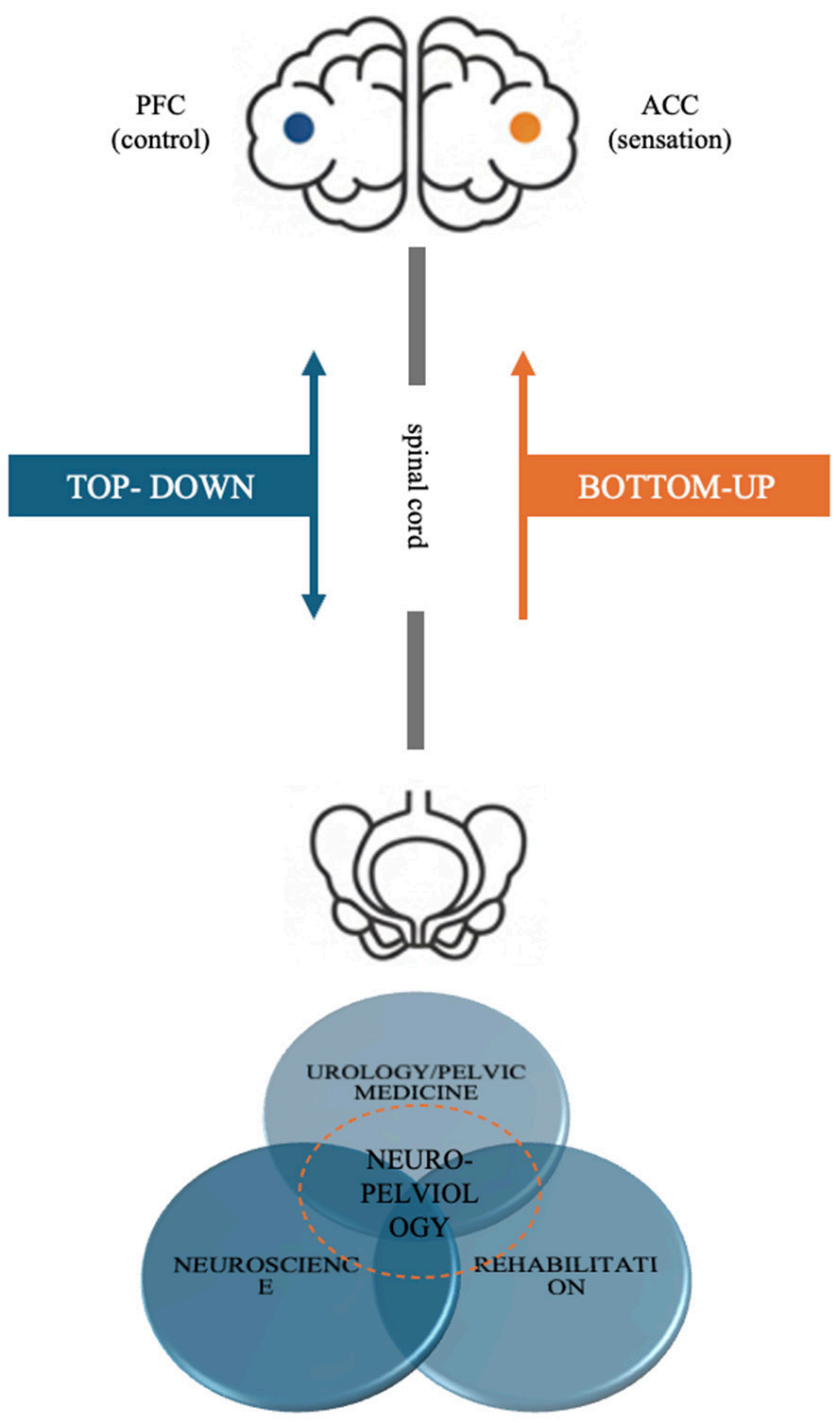
Conceptual framework of the cerebro-pelvic axis (CPA) and the neuro-pelviology interdisciplinary research agenda. This diagram establishes a conceptual framework for the cerebro-pelvic axis (CPA), a bidirectional system connecting the brain to the pelvic floor via “descending control” pathways (originating from the PFC) and “ascending interoceptive” pathways (projecting to the insula/ACC). This framework reframes clinical issues into two working failure-mode categories: “downstream pathway disruption” (central dysfunction, such as in Alzheimer's disease) and “bottom-up disturbance” (peripheral signals interfering with the central nervous system, such as OAB and selected SUI/mixed UI phenotypes). Finally, the diagram positions neuro-pelviology as an interdisciplinary research agenda integrating neuroscience, urology/pelvic-floor medicine, and rehabilitation medicine, aiming to support integrative phenotyping and mechanism-oriented translational study designs for CPA-relevant dysfunction patterns. The diagram is intended as a control-architecture mapping and does not imply shared etiology between the illustrated conditions.

The current medical system routinely treats higher brain dysfunction (such as AD and chronic altered states of consciousness) and pelvic floor dysfunction (such as SUI) as two distinct and unrelated conditions. However, in current care pathways, cognitive/neurobehavioral syndromes and lower urinary tract/pelvic floor symptoms are typically assessed and managed in separate clinical silos, often with non-overlapping outcomes and limited cross-referral; this separation can obscure shared control-circuit vulnerabilities and hinder mechanism-oriented phenotyping across conditions. To adequately describe this coupled phenomenon, we must first explore a working descriptive and analytic vocabulary. To this end, we introduce two linked working terms: the CPA and neuro-pelviology. The specific framework is detailed in Figure 1.

### 2.1 The cerebro-pelvic axis (CPA)

#### 2.1.1 Definitions

We define the CPA as a control-loop mapping framework that integrates neuromodulation, endocrine signaling, somatosensory input and psycho-emotional processing. Functionally, the CPA connects the cerebral cortex with subcortical networks (including the frontal-parietal executive network, limbic system and the insula), the integration centers of the brainstem and thalamus, the autonomic and somatic motor centers of the spinal cord (particularly the thoracolumbar and sacral nuclei), and ultimately the effectors in the pelvic floor region that execute physiological behaviors (bladder, urethra, pelvic floor muscles, anal sphincter and genital-related neuromuscular structures) (Jang et al., 2018; Schellino et al., 2020). This axis comprises two fundamental functional limbs. The first functional limb is the descending (output) control pathway, which governs top-down regulation. Higher brain centers integrate commands related to urination, urinary control, pelvic floor stability, and sexual behavior into actionable directives such as “permit/inhibit” or “immediate/delayed” commands that incorporate precise timing and contextual considerations before transmitting these signals downward (Tish and Geerling, 2020). The second functional limb is the ascending (afferent) interoceptive pathway, which coordinates bottom-up feedback signals. Multimodal interoceptive signals from the pelvic region, such as bladder fullness, urethral pressure, perineal tension, pain, or leakage awareness, are continuously transmitted to interoceptive centers in the brain such as the insula and anterior cingulate cortex. This process shapes subjective experiences such as urgency, safety or the perception of threat (Pang et al., 2022; Santoso et al., 2025).

Scope statement (CPA in this paper). In this hypothesis paper, we discuss the CPA primarily in the context of lower urinary tract (LUT) control and pelvic-floor outlet coordination—i.e., bladder storage–voiding regulation and urethral/pelvic-floor muscle synergies, indexed by urodynamics and pelvic-floor EMG. We adopt this restricted scope because human evidence and measurable brain–pelvis coupling paradigms are currently most mature for LUT phenotypes (e.g., AD/pDoC-associated incontinence, OAB, and SUI/mixed UI). Other pelvic phenotypes that may also map onto a broader brain–pelvis control loop—including dysfunctional voiding/functional outlet obstruction, bowel dysfunction, sexual dysfunction, and postpartum pelvic-floor syndromes are acknowledged as important extensions but are not systematically analyzed here due to limited mechanistic evidence and space constraints. The failure-mode framework (top-down disintegration vs. bottom-up disturbance) is therefore operationalized for urinary continence/voiding control, with the intent to extend it to other pelvic phenotypes as standardized biomarkers and synchronized paradigms become available.

We explicitly exclude common LUT phenotypes such as bladder outlet obstruction (BOO), including BPH-related obstruction, and urinary retention/underactive bladder from the current framework. These phenotypes, which involve increased outlet resistance, impaired detrusor contractility, attenuated afferent signaling, or mixed mechanisms, are not included in the present “top-down disintegration” and “bottom-up disturbance” failure-mode scheme, as they do not align clearly with the definitions of these categories. In this paper, they are treated as important extension cases for future refinement of CPA phenotyping rather than fully specified CPA categories.

#### 2.1.2 The CPA as a dynamic feedback loop

The CPA does not represent a unidirectional relationship in which the brain controls the pelvic floor but represents a real-time dynamic feedback loop that encodes peripheral physiological states into subjective experiences and translates these experiences into behavioral decisions. For instance, the mechanical stimulus created by “bladder fullness” is translated within the CPA framework into the behavioral command “I need to find a restroom,” which carries cognitive priority and the assessment of social consequence. Conversely, “holding urine” is not merely a simple contraction of the sphincter muscles, but rather a sustained inhibitory command issued by the prefrontal executive network to the spinal micturition reflex. At its core, the CPA represents a higher-order cognitive control strategy (Michels et al., 2015; Schott et al., 2023). This formulation implies measurable coupling (and decoupling) between central decision networks and peripheral physiology, enabling falsifiable biomarkers of control-loop integrity.

#### 2.1.3 Disease reconstruction under the CPA framework

The CPA provides a working integrative mapping framework that enables us to map different clinical conditions onto a shared control architecture for phenotyping purposes. Urinary incontinence in AD or pDOC may be mapped as a failure of the upstream control layer of the CPA (the cortico-thalamic-brainstem integration circuit). When the brain loses its capacity to inhibit, plan and time pelvic floor actions, voiding behavior regresses to a lower-level automated reflex (Sugimoto et al., 2017; Qin et al., 2021). Conversely, SUI is primarily an outlet/continence-mechanism failure at the effector level (Aoki et al., 2017). Within the CPA framework, it may be mapped as a pelvic-floor–dominant phenotype at the output end of the control loop. In selected subgroups (e.g., mixed UI or SUI with prominent urgency/distress), recurrent leakage and anticipatory fear may also be associated with secondary ascending salience-like signaling and cognitive-affective burden, which should not be generalized as a primary mechanism in pure SUI. In other words, the CPA framework is intended to complement, rather than replace the established mechanical model of SUI in phenotyped subgroups where symptom burden and psychosocial distress may contribute to clinical severity (Dutta and Pathak, 2024).

Rather than arguing for disease-level coupling, the CPA offers a control-architecture mapping in which AD/pDoC-related urinary dysfunction and pelvic-floor/LUT–dominant phenotypes (e.g., OAB and selected SUI/mixed UI presentations) may be mapped to dysfunction at different nodes of a shared brain–spinal–pelvic control loop. More specifically, the hypothesis concerns a reproducible control-loop mapping pattern—i.e., partially coupled clinical presentations that may reflect dysfunction in shared control nodes, computations, or vulnerability contexts—rather than a claim of shared pathology. For hypothesis generation and phenotyping, the CPA emphasizes three mapping dimensions that may be partially shared across otherwise distinct conditions: (i) control nodes (e.g., mPFC/ACC–PAG–PMC); (ii) control computations (e.g., executive inhibition and interoceptive/salience weighting); and (iii) overlapping vulnerability contexts (e.g., aging, cerebrovascular burden, medication effects, frailty). This is not a claim of mutual causality or shared pathology. Rather, it is a node-level mapping claim in which dysfunction at different levels of the same control loop may generate partially coupled phenotypic patterns, which should be treated as distinct failure-mode expressions within the CPA framework.

### 2.2 Neuro-pelviology

#### 2.2.1 Definition

In this work, we use “neuro-pelviology” to refer to a provisional interdisciplinary research agenda grounded in the CPA framework, which allows the systematic investigation of physiological structure, information flow, plasticity, and patterns of pathological dysregulation to develop diagnostic and interventional strategies. The knowledge system encapsulated by neuro-pelviology spans neuroscience, urology, gynecology, rehabilitation medicine, and bioengineering, but it is not limited to these specific fields. It is necessary to distinguish this broad and system-centered concept from the existing and more surgery-focused field of “neuropelveology” to avoid conceptual overlap (Fowler et al., 2008; Possover, 2011). Here, “neuropelveology” is used in its established sense as a clinically oriented discipline focusing on peripheral pelvic nerve pathology, often leveraging surgical exploration/reconstruction and pain-centric phenotyping. In contrast, we use “neuro-pelviology” to denote a systems-neuroscience and rehabilitation-facing research program in which the unit of analysis is the brain–spinal–pelvic control loop, the primary outputs are mechanism-oriented phenotypes (failure modes), and the core measurable readouts include synchronized neurophysiology/neuroimaging with urodynamics/EMG. The intent is not terminological novelty, but to define a translational scope that connects circuit models to falsifiable biomarkers and trial designs.

#### 2.2.2 The paradigm shifts in neuro-pelviology

Compared with conventional single-specialty perspectives, the CPA-guided neuro-pelviology research agenda offers three practical shifts in perspective. First, this concept adopts the central axis as the unit of measurement, treating the brain and pelvic floor as a single and malleable physiological entity. Second, by using failure modes as the unit of analysis, clinical dilemmas are redefined as classifiable faults at different levels of the CPA (e.g., upstream control breakdown vs. downstream execution instability). This provides a universal language for diagnosis and research targeting cross-disease mechanisms. These two approaches have precedent in other axis-oriented frameworks (e.g., the gut–brain–microbiome axis), where bidirectional dysregulation has been used to organize heterogeneous clinical syndromes. Bidirectional dysregulation within the same axis can simultaneously explain clinical syndromes that were previously categorized as distinct gastrointestinal disorders and neurological disorders. This suggests that problems should be described based on the specific dysfunctional pattern within the axis (i.e., identifying which segment is failing) rather than traditional disease labels. Consequently, this approach enables the investigation of common pathological mechanisms and therapeutic targets across diseases (Zhao et al., 2018). Ultimately, this working concept guided by a multi-level interventional approach that inherently supports an engineering-oriented objective: establishing a CPA-referenced, tiered and targeted individualized testable individualized intervention strategies (Panicker et al., 2015), Furthermore, different levels can be targeted by distinct neuromodulation tools, such as cortical networks (rTMS), spinal cord centers (rTSMS), and peripheral afferents (SNM) (Chen A. et al., 2024; Dequirez et al., 2025).

In this article, neuro-pelviology is used to outline a provisional interdisciplinary research framework and translational agenda for the interdisciplinary research agenda of functional interactions between the brain and pelvis.

## 3 The biological basis of the CPA: from concept to circuitry

In this section, we outline circuit-level mechanisms that render the CPA a measurable and testable control-loop mapping, with intervention-eligible nodes across cortical, brainstem, spinal, and peripheral levels. These circuits provide a mechanistic scaffold for interpreting clinical syndromes as directional failure modes of a single control loop. Much of the circuit-level evidence summarized here derives from animal, translational, and neuroimaging studies; therefore, species differences and inferential gaps should be considered when extrapolating to human clinical phenotypes.

### 3.1 The downstream control pathway: the command chain from intent to execution

For humans, urinary control is a quintessential goal-directed behavior that requires the simultaneous fulfillment of both physiological and social imperatives. This implies that the brain must coordinate decision-making across multiple higher-level modules and relay results to the pelvic floor. This multi-tiered and intricate chain of command ensures that functionality of the pelvic floor evolves from primitive reflexes into complex behaviors that are regulated by both conscious intent and situational context.

The starting point of the descending control pathway in the CPA is located in the cerebral cortex, specifically the prefrontal cortex (PFC) and anterior cingulate cortex (ACC). The PFC is responsible for executive control and situational judgment and inhibits inappropriate urges to urinate (Kuhtz-Buschbeck et al., 2005). The latter integrates a sense of urgency–risk perception to assess whether an individual can endure the sensation any longer (Gao et al., 2015). The insula translates signals from the pelvic region into subjective urinary urgency intensity, while emotional structures such as the amygdala assign emotional connotations of “threat” or “safety,” collectively determining whether to initiate the process of urination (Pang et al., 2022).

The integrated output from these cortical and subcortical regions first converges in the periaqueductal gray (PAG) of the brainstem (de Groat et al., 2015), which serves as a critical relay and gating hub to integrate ascending inputs and descending cortical commands (Kitta et al., 2015). When this integrated assessment yields a “permit urination” outcome, the PAG activates the pontine micturition center (PMC), which functions as a master switch and pattern generator for urination. The PMC translates abstract behavioral decisions into a precisely choreographed sequence of neural discharge patterns (de Groat and Wickens, 2013). Subsequently, this pattern projects via brainstem-spinal descending pathways to the thoracolumbar (T11–L2) and sacral (S2–S4) spinal nuclei. On one hand, the PMC inhibits sympathetic output mediating urine storage and the activity of the pudendal nerves originating from the Onuf nucleus, thereby releasing the urethral and pelvic floor sphincter defenses. In contrast, the PMC activates sacral parasympathetic nerves to drive detrusor contraction, achieving efficient and coordinated micturition at the peripheral level (Stoffel, 2016; Kim et al., 2020).

This descending pathway is not merely a simple regulatory mechanism, but rather a central dispatch system that compresses social judgments, emotional appraisals, autonomic outputs and striated muscle precision contractions into a single behavioral scheme. This may help interpret why urinary dysfunction in AD or pDoC often co-presents with cognitive or consciousness impairments, potentially representing systemic damage to the upstream control layers of the CPA.

### 3.2 The ascending sensory pathway: constructing perception from signals

The ascending pathways of the CPA transmit real-time physiological signals from the pelvic floor to the brain and contribute to subjective sensation and behavioral responses.

Mechanoreceptors, chemoreceptors, and nociceptors in the pelvic region encode a range of signals, including distension, stretching and pain. These signals are transmitted via the pelvic nerves and pudendal nerves to the dorsal horn of the sacral spinal cord (de Groat and Yoshimura, 2009). Subsequently, the signal ascends along pathways such as the spinothalamic tract, relays through the parabrachial nucleus, and projects to the thalamus, the gateway through which sensory information enters the cerebral cortex (Pang et al., 2022). Ultimately, the signal reaches the insular cortex and ACC, the two core components of the interoceptive hub (Ketai et al., 2016). Here, the original neural signals are translated into subjective experiences we can perceive, such as “a slight urge to urinate,” “a strong sense of urgency” or “discomfort in the pelvic floor.”

The function of this pathway extends beyond the mere conveyance of physiological states. Persistent abnormal sensory input is most clearly established in pain-dominant conditions such as chronic pelvic pain (Till et al., 2019). In LUT disorders such as OAB and in selected SUI/mixed UI subgroups with prominent urgency or leakage-related distress, symptom monitoring and anticipatory vigilance may also increase cognitive and emotional burden. The magnitude and mechanisms of these effects likely differ across phenotypes and should not be assumed equivalent. Such interference may consume executive resources, including attention and working memory, that are managed by prefrontal control systems, thereby contributing to impaired performance in other cognitive tasks (Steiner et al., 2020).

The ascending interoceptive pathway may do more than report physiological states; in some phenotypes, persistent symptom-related input may shape emotional baseline, attentional allocation, and self-perception over time. Pelvic floor disorders may, in some phenotypes, be associated with changes in central processing and network organization via ascending pathways of the CPA.

### 3.3 Neurotransmitters: the chemical language of the CPA

The integrative function of the CPA relies not only on anatomical connections but also on a shared chemical language between the central and peripheral nervous systems. This language is mediated by norepinephrine (NE), 5-hydroxytryptamine (5-HT), dopamine (DA) and acetylcholine (ACh), which play characteristic dual roles at different levels of the CPA.

**Figure 2:**
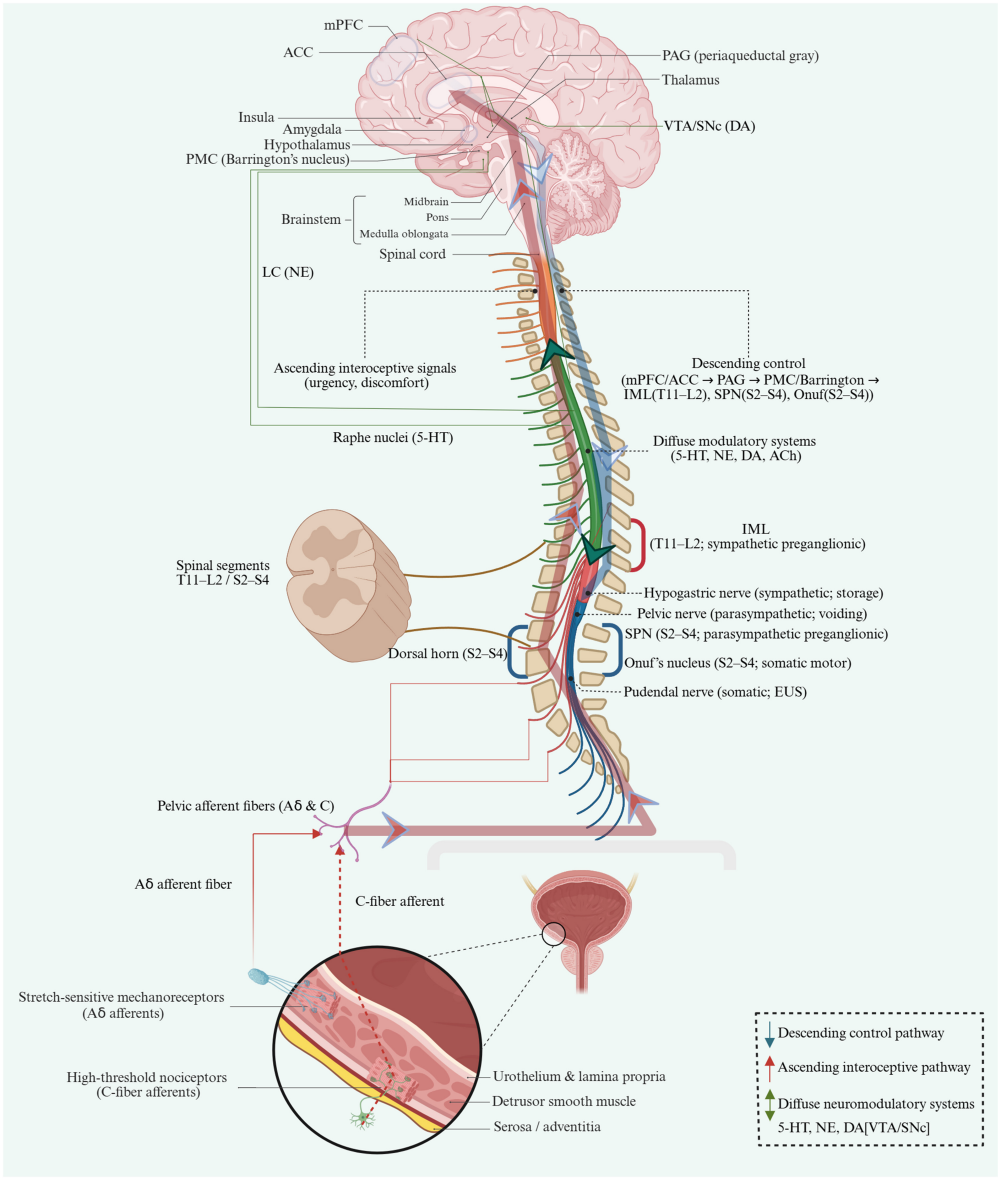
Established brain–spinal–peripheral circuits and neuromodulatory systems supporting the cerebro-pelvic axis (CPA). This schematic summarizes established neurourological control pathways linking supraspinal networks with spinal autonomic and somatic outflow that coordinate lower urinary tract (LUT) storage and voiding. Ascending interoceptive signals originate from bladder mechanosensitive Aδ afferents and high-threshold C-fiber afferents, enter the spinal cord (including the sacral dorsal horn, S2–S4), and engage supraspinal relay and integration regions including the periaqueductal gray (PAG) and thalamus, with cortical processing in interoceptive–salience regions (e.g., insula, ACC) contributing to urgency/discomfort perception. Descending control is conveyed from cortical control regions (e.g., mPFC/ACC) to the PAG and the pontine micturition center (PMC/Barrington's nucleus), which in turn coordinates spinal outputs via the intermediolateral cell column (IML, T11–L2; sympathetic preganglionic), sacral parasympathetic nucleus (SPN, S2–S4; parasympathetic preganglionic), and Onuf's nucleus (S2–S4; somatic motor). Peripheral efferent pathways are indicated as the hypogastric nerve (predominantly sympathetic; storage), pelvic nerve (predominantly parasympathetic; voiding), and pudendal nerve (somatic; external urethral sphincter control). Diffuse neuromodulatory systems (e.g., 5-HT from raphe nuclei, NE from locus coeruleus, DA from VTA/SNc, and cholinergic systems) are shown as widespread modulators that shape arousal, affective state, and network excitability across these circuits. ACC, anterior cingulate cortex; mPFC, medial prefrontal cortex; PAG, periaqueductal gray; PMC, pontine micturition center; IML, intermediolateral cell column; SPN, sacral parasympathetic nucleus; EUS, external urethral sphincter.

The noradrenergic system elevates urethral smooth muscle tone via sympathetic pathways in the spinal cord and periphery, serving as a crucial pharmacological basis for maintaining the threshold for urinary retention during the storage phase (Andersson and Uvelius, 2024). In the central nervous system, the noradrenergic system, originating from the locus coeruleus, regulates alertness and threat monitoring, playing a crucial role in the interoceptive vigilance state that determines whether maintaining pelvic floor closure must be prioritized (Ross and Van Bockstaele, 2020). Dysfunction of the noradrenergic system may couple stress-related hypervigilance with symptoms in the lower urinary tract, including urgency and frequency. The 5-HT system also exhibits central-peripheral parallel regulation. At the level of the spinal Onuf nucleus, 5-HT enhances excitability of the external urethral sphincter motoneurons, forming the primary pharmacological basis for the administration of duloxetine to treat SUI (Jost and Marsalek, 2004); At the brain level, 5-HT plays a critical role in regulating mood and impulse control (da Cunha-Bang and Knudsen, 2021). This imbalance may be associated with reduced emotional tolerance and altered sphincter-control thresholds; however, in most human data the relationship is bidirectional or associative. We therefore treat 5-HT as a candidate coupling mechanism within the CPA loop to be tested, rather than assuming a direct causal chain. As a key neurotransmitter in the basal ganglia-cortex motor and motivational circuitry, dopamine not only participates in general motor planning and behavioral selection but also modulates inhibition of the PMC via the striatum-thalamus-cortex circuit (Wang et al., 2020); In untreated patients with early-stage Parkinson's disease (PD), lower urinary tract symptoms are closely associated with dopaminergic degeneration in the substantia nigra-striatal pathway, thus indicating that dysfunction of the DA system can translate into a phenotype of detrusor overactivity and urgency/urge incontinence via the descending CPA pathway. Acetylcholine provides another key link between the CPA, consciousness and cognition. Peripherally, cholinergic parasympathetic efferents serve as the primary pathway driving detrusor contraction and initiating the micturition reflex (Fowler et al., 2008); In the central nervous system, the basal cholinergic systems in the forebrain and brainstem serve as pivotal hubs for maintaining wakefulness, attention and cortical activation. The functional decline of these systems aligns closely with key pathologies of AD and pDoC (Bekdash, 2021; Raciti et al., 2023) and offers a candidate control-loop coupling mechanism that may contribute to the co-presentation of cognitive/consciousness decline and urinary incontinence in some cohorts, although much of the current evidence is indirect and associative. Figure 2 provides an integrated overview of these descending/ascending neural circuits and their corresponding diffuse neurotransmitter systems to explore a structural foundation for considering the CPA as a measurable and intervention-eligible control-loop mapping.

In summary, although the individual components of the CPA have been extensively investigated across various disciplines, the originality of the CPA framework lies in integrating these components into one system with a clear functional division of labor (downward control/upward interoception) and hierarchical structure, emphasizing the dynamic interactions of this axis as a unified system in both healthy and disease states. The clinical dysregulation described hereafter relates precisely to the manifestation of a system-level control-loop dysfunction.

## 4 Cross-disciplinary clinical evidence for CPA-relevant control-loop dysfunction patterns

We performed extensive literature reviews and have compiled clinical evidence from multiple research areas, including neurology, rehabilitation medicine, and urology, to demonstrate that multiple seemingly unrelated diseases can in fact be considered as functional impairments of the central nervous system at different levels and in different directions. For this hypothesis paper, we use two-category working classification (top-down disintegration and Bottom-Up Disturbance) to organize the currently most developed examples. We recognize that some LUT phenotypes, particularly urinary retention/underactive bladder and outlet-obstructive conditions may involve mixed, attenuated-signal, or reduced-drive mechanisms that are not fully captured by this provisional scheme.

### 4.1 Top-down disintegration: the pelvic floor phenotype in central nervous system disorders

The descending control pathways may also malfunction when lesions occur in the upstream control centers of the CPA (the cerebral cortex, subcortical structures and brainstem). Within the CPA framing, this dysfunction may be clinically informative as a manifestation of upstream control-loop decompensation, rather than being treated solely as a late-stage care complication. We describe this phenomenon as a descending CPA disconnection pattern that is characterized by disruption in the upstream command chain that initiates dysfunctionality in the pelvic floor execution system (Tish and Geerling, 2020).

#### 4.1.1 AD and pDoC

The onset of urinary incontinence in AD is highly synchronized with declines in executive function and the capacity for behavioral inhibition (Sugimoto et al., 2017; Griebling, 2018). This aligns closely with disruption of the PMC control circuit within the CPA descending pathway. Similarly, in patients with pDoC, the widespread loss of urinary control may reflect a clinically relevant manifestation of concurrent disruption between the consciousness network and the CPA control network (Leboutte et al., 2023). In both scenarios, the loss of pelvic floor control is not merely a late-stage nursing detail or a simple decline in self-care abilities, but rather a core neurological symptom of dysfunction within the upstream integrative network of the central nervous system.

#### 4.1.2 PD and stroke

Overactive bladder symptoms in PD are often multifactorial, reflecting varying contributions from dopaminergic–basal ganglia dysfunction, autonomic changes, medications, and comorbid peripheral factors. Within the CPA framework, PD can therefore express mixed failure modes, and phenotyping should prioritize axis-level readouts (urodynamics/EMG plus central biomarkers) over a single-node explanation (Sakakibara et al., 2010). In stroke, LUT dysfunction often shows temporal evolution (e.g., early hypocontractility/retention followed by later overactivity), which fits a control-loop view in which different CPA nodes recover or decompensate over time. Accordingly, CPA phenotyping should be time-stamped and ideally longitudinal, rather than purely lesion-location–based (Khan et al., 1990). Detrusor-sphincter dyssynergia is known to be caused by lesions in the brainstem (Sakakibara et al., 1996). Compared to traditional neurogenic bladder classifications, the CPA perspective places greater emphasis on the phenotypic differences corresponding to damage at different nodes along the descending pathways.

### 4.2 Bottom-up disturbance: putative central network adaptations in selected pelvic phenotypes with persistent symptom-related afferent burden

When primary dysfunction occurs downstream of the CPA, persistent symptom-related afferent signaling in selected phenotypes (most clearly pain-dominant or urgency-predominant conditions, and some mixed UI/urgency-distress SUI subgroups) may influence central processing via ascending pathways and has been associated with alterations in central neural networks. Recent rs-fMRI studies report group-level differences in interoceptive–salience networks (often centered on the insula/ACC) and their connectivity with frontoparietal executive networks in OAB. In some cohorts, these measures correlate with symptom severity (e.g., urgency/incontinence), supporting—but not proving—the hypothesis that persistent peripheral afferent input could contribute to central network adaptations (Mehnert et al., 2023). We describe this phenomenon as a putative axis-level maladaptive state (here provisionally referred to as “CPA circuitopathy” for descriptive convenience), referring to putative axis-level dysregulation in which aberrant ascending signaling is hypothesized to be linked with maladaptive central network plasticity. This concept does not aim to explore a new clinical syndrome, but rather to emphasize how peripheral dysfunction induces systemic alterations in central networks via maladaptive neuroplasticity.

#### 4.2.1 SUI and OAB

Pure SUI is primarily an outlet/continence-mechanism failure under increased abdominal pressure. In addition to peripheral mechanical defects, the persistent fear of urinary leakage experienced by a subset of patients with SUI—particularly those with mixed UI, coexisting urgency, or high symptom-related distress—may become a subgroup-specific salience-like signal. This signal may be associated with increased engagement of brain regions related to symptom monitoring, anxiety, and vigilance (such as the insula and ACC) via the ascending CPA pathway, potentially increasing executive control demands in the PFC (Di Gangi Herms et al., 2006). Neuroimaging studies suggest that a subset of OAB patients may exhibit altered salience-network processing consistent with “misinterpretation” and “amplification,” leading the brain to prematurely and excessively interpret these as urgent signals (Ketai et al., 2016, 2021). However, we note that rs-fMRI findings can be heterogeneous across cohorts and analytic pipelines; thus, these interpretations should be considered probabilistic rather than definitive. In summary, selected SUI phenotypes (especially mixed UI or urgency/distress-prominent subgroups) may be provisionally framed as bottom-up–disturbance–relevant presentations, whereas pure SUI remains primarily an outlet/mechanics phenotype. In pure SUI, any central changes should be interpreted cautiously as potential secondary correlates (if present) rather than core mechanisms. We acknowledge that some patients may exhibit mixed or shifting phenotypes (e.g., combined impaired inhibition and heightened afferent salience), and the CPA framework is intended to accommodate such cases as hybrid failure modes rather than forcing a binary classification.

#### 4.2.2 Chronic pelvic pain (CPP)

CPP may provide one of the clearest clinical examples of central nervous system involvement associated with persistent ascending symptom input by dysfunction in the ascending pathways of the CPA. Long-term and persistent pelvic pain signals have been shown to induce significant structural and functional changes in the brain; this condition is known as central sensitization. Pain, emotion, and cognition-related networks (such as the default mode network) all exhibit abnormalities in functional connectivity, thus providing a plausible neurobiological basis for the frequent co-occurrence of CPP with depression, anxiety, and cognitive impairment (As-Sanie et al., 2016). Furthermore, compared to the interoceptive disturbances in SUI/OAB that are characterized by urgency and the fear of leakage, CPP manifests more as persistent pain-driven alterations in affective-cognitive networks.

Synthesizing the above evidence, we arrived at an interdisciplinary conclusion in that AD, pDoC and similar conditions represent disintegrative failures of the descending pathways of the CPA, while OAB, CPP, and selected SUI/mixed UI phenotypes with prominent urgency/distress may represent bottom-up disturbance patterns involving ascending CPA pathways. In many complex cases, particularly among the elderly or critically ill patients undergoing rehabilitation, these two failure modes often coexist to form a vicious cycle of bidirectional loss of control. Within the CPA framework, these failure modes are clearly defined as distinct dysregulation phenotypes along the same control architecture, as distinct dysregulation phenotypes mapped to different nodes (or mixed states) within a shared control architecture, rather than as an etiologic coupling claim. Table 1 systematically summarizes representative diseases, pelvic floor phenotypes, and hypothesized mechanisms under different CPA failure modes, thus providing a foundation for investigating integrated and mechanism-oriented interventional strategies.

**Table 1:** Clinical evidence supporting the CPA framework (non-exhaustive examples within the current working taxonomy).

**CPA failure mode**	**Cohort context**	**Pelvic floor function**	**Relevant CPA mechanisms**	**References**
Descending pathway disintegration	Alzheimer's disease (AD)	Urge incontinence, frequent urination, nocturia	Frontal–limbic degeneration may reduce inhibitory control over brainstem micturition circuitry (PAG/PMC) and is associated with co-occurring LUT symptoms. Cholinergic deficits may be a plausible coupling mechanism, but current human evidence is predominantly associative.	Panicker, 2020; Chang et al., 2021
Descending pathway disintegration	Traumatic brain injury (TBI)	Complete urinary incontinence often requires indwelling catheterization	Damage to rostral/corticothalamic–brainstem pathways may compromise supraspinal control and is associated with loss of voluntary continence in severe cases. The relative contribution of spinal reflex dominance vs. peripheral/iatrogenic factors is likely cohort- and phase-dependent.	Albayram et al., 2019
Descending pathway disintegration	Parkinson's disease (PD)	Detrusor overactivity → Urgency/Urge incontinence	Nigrostriatal dopaminergic degeneration may reduce inhibitory modulation of brainstem micturition control (PAG/PMC) and is associated with DO/OAB-like phenotypes. Mechanistic contributions are multifactorial (autonomic changes, medications, comorbidities), supporting a phenotyping-first interpretation.	Wang et al., 2020; Roy et al., 2018; Kim et al., 2024
Descending pathway disintegration	Stroke	Most cases involve urge or mixed urinary incontinence; some lesions may cause urinary retention	Lesions affecting frontal–subcortical or brainstem pathways may disrupt descending inhibitory control and storage–voiding coordination. LUT phenotypes often evolve over time (including mixed UI or transient retention), supporting time-stamped control-loop phenotyping rather than single-mechanism attribution.	Agapiou et al., 2024
Bottom-up disturbance (selected pelvic phenotypes)	Selected SUI/mixed UI phenotypes (urgency- or distress-prominent; not pure SUI broadly)	Stress leakage (e.g., exertion/cough), often with coexisting urgency and/or high leakage-related distress in selected subgroups	Pure SUI is primarily an outlet/mechanical phenotype. In carefully phenotyped mixed UI or urgency/distress-prominent SUI subgroups, recurrent leakage-related distress may increase symptom vigilance and psychosocial burden, with central changes interpreted cautiously as secondary correlates rather than core mechanisms of pure SUI. PFM-related cortical activation/plasticity is treated as compensatory or training-related rather than a core mechanism of pure SUI.	Di Gangi Herms et al., 2006; Li and Wang, 2024
Bottom-up disturbance (selected pelvic phenotypes)	Overactive bladder (OAB)	Urinary urgency (urge incontinence), increased frequency, nocturia	Some cohorts show altered interoceptive–salience network engagement (insula/ACC) during filling/urgency and variable prefrontal control signatures. Findings are heterogeneous across cohorts and analytic pipelines and should be interpreted as probabilistic, hypothesis-supporting associations rather than established causal adaptations.	Zuo et al., 2019
Bottom-up disturbance (selected pelvic phenotypes)	Chronic pelvic pain (CPP)/Interstitial Cystitis (IC)/Bladder Pain Syndrome (BPS), etc.	Persistent pelvic pain, often accompanied by frequent urination/pain during bladder filling	Persistent pelvic pain is associated with central sensitization and altered affective–cognitive network organization (e.g., salience/default-mode circuitry). These observations support bottom-up–disturbance relevance in pain-dominant phenotypes, while causal directionality remains to be established.	Kutch et al., 2017; Clemens et al., 2019

## 5 Targeted approaches: a new generation of intervention strategies

Reconstructing clinical problems as patterns of CPA malfunction can provide us with more precise and mechanism-based treatments. On this basis, we outline a conceptual, hypothesis-generating intervention paradigm, which we term coordinated axis neuromodulation (CAN).

### 5.1 Neuromodulation: the precision toolkit for neuro-pelvic science

Neuromodulation technologies offer promising interventions targeting different levels of the CPA, enabling us to modulate multiple nodes of the brain–spinal–pelvic control loop from cortex through spinal cord to periphery.

#### 5.1.1 Central targeting: repairing the “command headquarters”

The core objective of central targeting is to restore inhibitory-permissive functionality in the brain. Current research focuses on the dorsolateral prefrontal cortex (DLPFC) to enhance executive control (Li Y. et al., 2023); and the supplementary motor area (SMA) to enhance pelvic floor motor planning and feedforward control (Groenendijk et al., 2020), Repetitive transcranial magnetic stimulation (rTMS) has been widely employed to modulate cortical networks, with the rationale for relatively standardized stimulation protocols. Standardized high-frequency rTMS over the DLPFC and SMA has been reported to be feasible and associated with preliminary efficacy signals across multiple domains (Yani et al., 2019; van Eijndhoven et al., 2020). In clinical trials for CPA-related disorders, a 4-week course of rTMS combined with a multimodal assessment system, including clinical indicators such as visual analog scales for urinary urgency and incontinence diaries, alongside neuroimaging biomarkers such as resting-state/task-state fMRI, has been used in studies relevant to components of the proposed CPA framework (Chen J. et al., 2024). Furthermore, the combined TMS-EEG technique is being explored as a potential electrophysiological biomarker for evaluating changes in cortical excitability (Noda et al., 2024).

#### 5.1.2 Spinal targeting: tuning the “relay station”

The core objective of spinal cord targeting is to modulate spinal reflex circuits and improve detrusor-sphincter coordination. The primary techniques include non-invasive repetitive transspinal magnetic stimulation (rTSMS) and invasive spinal cord stimulation (SCS). These two techniques enable bidirectional regulation of the storage and voiding of urine by segmental and frequency modulation. Targeting the thoracolumbar segment (T11–L2) is hypothesized to favor sympathetic-mediated storage function and may be associated with increased bladder compliance, whereas targeting the sacral segment (S2–S4) may modulate parasympathetic and somatic circuits relevant to voiding coordination (Steadman and Grill, 2020; Sysoev et al., 2024).

Non-invasive rTSMS and invasive SCS can bidirectionally modulate storage and voiding functions, with small-scale studies reporting improved bladder capacity and sphincter coordination (Niu et al., 2018; Steadman and Grill, 2020; Zhang et al., 2022). These effects are typically quantified by urodynamic and EMG parameters.

#### 5.1.3 Peripheral targeting: reshaping the “information source”

The core purpose of peripheral targeting is hypothesized to reduce symptom-related afferent burden and modulate peripheral-to-central signaling, potentially diminishing excessive vigilance in ascending pathways in selected phenotypes. The primary methods used for peripheral targeting are sacral nerve modulation (SNM) and percutaneous tibial nerve stimulation (PTNS).

With long-term prospective studies supporting durable clinical benefit (Blok et al., 2020), during SNM, functional neuroimaging studies (e.g., fNIRS) have detected real-time alterations in the brain activity of OAB patients, thus providing preliminary imaging observations that are consistent with (but do not establish) the CPA hypothesis of peripheral-to-central coupling (Luo and Liao, 2025).

PTNS, as a low-cost and non-invasive alternative, may offer bottom-up neuromodulatory input to sacral circuits. Irrespective of whether we consider SNM or PTNS, the systems used to evaluate their clinical efficacy are becoming increasingly standardized, commonly employing a standardized 3-day bladder diary and validated symptom/quality-of-life scales (such as the ICIQ-UI-SF) for quantification (McClurg et al., 2022; Stanley et al., 2024).

### 5.2 CAN: an integrative new paradigm for treatment

Based on the CPA, we propose Coordinated Axis Neuromodulation (CAN), a testable paradigm of time-locked multi-node stimulation intended to produce incremental changes in pre-specified axis-coupling endpoints (central biomarkers and pre-registered peripheral endpoints, e.g., EMG timing indices and/or diary-defined urgency/UUI burden; urodynamics as supportive phenotyping/secondary mechanistic data when included) beyond dose-matched single-node neuromodulation. To avoid confusion with the established term central autonomic network (also abbreviated CAN), we use “CAN” here strictly to denote Coordinated Axis Neuromodulation within the CPA framework. These proposals are hypothesis-generating and require empirical validation in specifically designed studies.

CAN is presented solely as a falsifiable research construct rather than a recommended clinical protocol. The primary hypothesis is that temporally coupled multi-node stimulation produces measurable, direction-specific changes in axis-level (control-loop–level) coupling metrics [central markers ↔ pre-registered peripheral endpoint(s), e.g., pelvic-floor EMG timing indices and/or diary-defined urgency/UUI burden; urodynamics as supportive/secondary data when included] beyond what is achieved by any single-node stimulation matched for dose and contact time. The null hypothesis is that multi-node coupling provides no incremental change in these coupling metrics or clinical readouts compared with single-node stimulation.

To operationalize these hypotheses, axis-level coupling metrics may be defined as synchronized brain–pelvis associations between central markers (e.g., PFC/ACC/insula activity, connectivity indices, or TMS-EEG measures) and peripheral readouts. Depending on the hypothesized failure mode, peripheral readouts may include clinically meaningful endpoints (e.g., diary-defined urgency/UUI burden and validated symptom/QoL scales) and/or dynamic coordination measures (e.g., pelvic-floor EMG timing patterns), with urodynamics (UDS) treated as supportive phenotyping/secondary mechanistic data when included. Importantly, conventional urodynamic shifts (e.g., changes in DO or static cystometric parameters) are not required for CPA/CAN-consistent clinical benefit, particularly in scenarios where the hypothesized mechanism is executive/timing modulation rather than detrusor physiology *per se*, and OAB may be present without demonstrable DO on UDS. Accordingly, falsification should not be framed as a default “symptom improvement without UDS change,” but should be based on pre-specified primary coupling/biomarker endpoints appropriate to the failure mode, while treating UDS as supportive or phenotyping information when included. Prespecified stratification may classify cohorts as top-down–dominant or bottom-up–dominant using combined clinical and physiological markers. Falsification would be supported if temporally coupled multi-node stimulation does not produce the predicted direction-specific changes in the pre-registered primary coupling metric(s) or subgroup-pattern differences beyond dose-matched single-node stimulation. To make this framework operationally testable and to avoid *post hoc* interpretation, we provide pre-specified illustrative examples of coupling metrics, stratification rules, and falsifiers in Box 1.

Box 1 **Pre-specified examples for operationalization, stratification, and falsification of CPA/CAN predictions.**

These examples are intended to demonstrate how CPA/CAN predictions can be pre-specified for testing. General note (interpretation and falsifiability). In LUT disorders, clinically meaningful improvement can occur without detectable changes in DO or other conventional UDS parameters, and OAB phenotypes may be present without demonstrable DO on UDS. Therefore, UDS should be treated as supportive phenotyping and/or secondary mechanistic evidence when included, rather than a necessary gatekeeper for CPA/CAN-consistent benefit. Depending on the hypothesized failure mode, primary confirmatory/falsification logic may rely on (i) pre-registered clinical endpoints (bladder diary outcomes; validated symptom and QoL scales); (ii) dynamic coordination readouts (e.g., pelvic-floor EMG timing patterns); and/or (iii) central biomarkers (EEG/TMS-EEG/fNIRS/fMRI), in addition to or instead of UDS.

Example 1. Top-down–dominant prediction (AD/pDoC-associated LUT dysfunction)

(i) Coupling metric definition:

Central marker: prefrontal inhibitory control (PFC/ACC activation or TMS-EEG prefrontal excitability). Peripheral readout: dynamic coordination indices such as pelvic-floor EMG during suppression/voiding and time-stamped bladder diary outcomes (e.g., urgency burden, UUI episodes), with UDS storage-phase indices (e.g., urgency-threshold volume) included as supportive/phenotyping data when feasible. Coupling is quantified by the within-subject association between central markers and the pre-registered primary peripheral endpoint(s) (e.g., pelvic-floor EMG timing indices and/or diary-defined urgency/UUI burden); UDS indices, when included, are treated as supportive phenotyping/secondary mechanistic readouts. The predicted direction: stronger prefrontal inhibitory markers correlate with improved continence-control coordination indexed by EMG timing patterns and/or diary-defined outcomes; urodynamic shifts, if present, are supportive rather than required.

(ii) Provisional stratification rule:

Classify as top-down–dominant if clinical phenotype suggests central network impairment (e.g., AD/pDoC), and baseline evidence shows impaired executive markers with LUT dysfunction, not primarily driven by peripheral afferent burden.

(iii) Falsifiers beyond symptom change:

Refute (or revise) the mechanistic prediction if temporally coupled stimulation fails to change the pre-registered primary coupling metric(s) in the predicted direction or is not superior to dose-matched single-node stimulation. Symptom improvement without conventional UDS change is non-confirmatory for a urodynamic mechanism, not a default refutation of CPA/CAN.

Example 2. Bottom-up–dominant prediction (OAB or mixed UI/urgency-distress phenotypes)

(i) Coupling metric definition:

Central marker: salience/interoceptive network activation (insula/ACC) during urge states. Peripheral readout: bladder diary outcomes capturing urgency/UUI burden and dynamic coordination measures (e.g., pelvic-floor EMG co-contraction/timing), with UDS markers treated as supportive or baseline phenotyping data when available (not required to demonstrate clinical benefit). Coupling is quantified by the association between central salience markers and peripheral indices. The predicted direction: excessive salience weighting correlates with maladaptive EMG patterns, and successful CAN/PTNS/SNM protocols reduce this maladaptive coupling.

(ii) Provisional stratification rule:

Classify as bottom-up–dominant when persistent urgency/distress is prominent, and baseline central salience/interoceptive markers are elevated, without pure outlet-mechanical SUI symptoms.

(iii) Falsifiers beyond symptom change:

Refute (or require revision of) this mechanistic prediction if the pre-registered primary salience/interoceptive marker fails to show the predicted change in coupling with the chosen primary peripheral endpoint (e.g., diary-defined urgency/UUI burden or EMG timing indices), or if stratified cohorts fail to show the predicted differences. Lack of change in DO or other conventional UDS parameters should not be treated as a default falsifier, given known limitations in UDS sensitivity and the possibility of OAB without demonstrable DO.

Example 3. Cross-failure-mode discrimination prediction (predictive value of the axis framing)

(i) Coupling metric definition:

Composite panel: one top-down coupling metric (PFC/TMS-EEG ↔ dynamic coordination indices such as pelvic-floor EMG timing and/or diary-defined continence control) and one bottom-up coupling metric (insula/ACC ↔ diary-defined urgency/UUI burden and/or EMG co-contraction patterns), with UDS included as supportive phenotyping or secondary mechanistic evidence when available. Coupling is quantified separately for each metric using standardized z-scores.

(ii) Provisional stratification rule:

Participants are categorized into top-down–dominant, bottom-up–dominant, mixed, or unclassified groups using pre-registered cut points based on the two coupling metrics and clinical phenotype.

(iii) Falsifiers beyond symptom change:

Refute if the coupling panel does not improve prospective prediction of mechanism-relevant responses (e.g., central-targeting vs. peripheral-targeting) beyond symptom-based models.

**Practical note:** These examples illustrate the logic for pre-specification. Specific biomarkers, acquisition platforms, and statistical models should be pre-registered and adapted to cohort feasibility.

The core concept of CAN is to synchronously target the cortex–spinal cord–peripheral triad within a single treatment session, with the hypothesis that temporally coordinated stimulation may yield synergistic effects at the axis level by combining upstream inhibitory support, relay-level reflex-threshold modulation, and downstream/peripheral afferent-burden modulation. For the purpose of hypothesis testing, future CPA/CAN studies should pre-register one primary coupling metric, one pre-specified stratification rule, and one falsifier beyond symptom change before enrollment, and should treat symptom-only improvement without predicted coupling change as non-confirmatory for the CPA/CAN mechanism claim.

The core concept of CAN is to synchronously target the cortex–spinal cord–peripheral triad within a single treatment session, with the hypothesis that temporally coordinated stimulation may yield synergistic effects at the axis level by combining upstream inhibitory support, relay-level reflex-threshold modulation, and downstream/peripheral afferent-burden modulation.

First, the principle of temporal coupling hypothesizes that temporal coupling may facilitate through the precise control of temporal intervals between stimuli delivered to different nodes. Basic research has indicated that when applied simultaneously to the cortex and spinal cord, paired associative stimulation can alter the excitability of both structures within a precise temporal window. As proof-of-concept neuromotor evidence, sequentially coupling paired stimulation with rehabilitation training has been shown to enhance motor output in selected contexts (Dixon et al., 2016; Pulverenti et al., 2022), supporting the plausibility of time-dependent multi-node plasticity rather than direct LUT-specific clinical translation. Second, the principle of closed-loop signaling could involve the dynamic adjustment of stimulation parameters based on real-time physiological or neurobiological feedback. Preliminary work indicates feasibility, but clinical scalability remains uncertain. For example, studies have employed wireless bladder pressure sensors or neural recordings from dorsal root ganglia to decode bladder status in real time, thereby triggering neuromodulation automatically. This approach has been reported to be potentially more energy efficiency and stimulation precision when compared to traditional continuous stimulation modes (Majerus et al., 2021; Ouyang et al., 2022). However, the clinical applicability of this new approach still requires further validation. Finally, the principle of individualized classification advocates for the selection of primary intervention targets based on a patient's specific CPA failure mode (e.g., descending disintegration vs. ascending hypersensitivity), may help shift broad-spectrum treatment to precision regulation. This concept has been repeatedly emphasized and discussed within research networks such as LURN and ICI-RS (Cameron et al., 2024; Finazzi Agrò et al., 2024).

To translate the CAN concept into actionable clinical research pathways, we next describe two illustrative prototype protocols that are aligned with distinct CPA failure modes: Prototype 1 and Prototype 2, these prototypes are presented as falsifiable study designs rather than recommended clinical protocols.

**Prototype 1:** AD with urinary incontinence (top-down disintegration dominant). This multimodal strategy aims to simultaneously restore impaired executive control and improve urinary storage. Facilitative rTMS/iTBS over the left dorsolateral prefrontal cortex (DLPFC) is used with the aim of engaging executive and inhibitory networks implicated in AD, in line with evidence arising from double-blind randomized controlled trials (Wu et al., 2022). In parallel, rTMS/rTSMS targeting the upper lumbar spinal segments (approximately T11–L1) is applied with the aim of modulating sympathetic-related storage function and bladder compliance, as suggested by limited human physiological studies (Yani et al., 2019); PTNS is incorporated as a peripheral adjunct to alleviate overactive bladder-like symptoms and potentially modulate symptom-related afferent input (Lashin et al., 2021). The synergistic effects of this combined and CPA-informed protocol for AD + UI remain hypothetical and require validation in specifically designed clinical trials.

**Prototype 2:** Selected SUI/mixed UI with urgency-distress burden (bottom-up disturbance relevance on an outlet-mechanical background). This approach focuses on strengthening central feedforwards control, optimizing pelvic floor muscle performance, and addressing maladaptive symptom salience and urgency-related afferent processing in carefully phenotyped patients (e.g., mixed UI or SUI with prominent urgency/distress), while recognizing that pure SUI is primarily an outlet-mechanics phenotype. Excitatory rTMS over the SMA is employed to facilitate cortical engagement and pre-activation of pelvic floor musculature, consistent with preliminary evidence that SMA-targeted stimulation can bidirectionally modulate the tone of the pelvic floor and related fMRI activity (Yani et al., 2019). This central intervention is combined with pelvic floor muscle biofeedback-guided training (PFM-BF) to refine peripheral muscle recruitment and skill acquisition, which has shown to provide additional benefits over PFMT alone in randomized trials (Wang et al., 2024). PTNS can be added as a further bottom-up neuromodulatory component to reduce urgency and improve bladder diary outcomes in patients with coexisting storage symptoms (Li X. et al., 2023). As with Prototype 1, this integrated protocol should be regarded as a conceptual template that requires empirical testing rather than a ready-to-use clinical recipe.

A future CPA-guided intervention framework may incorporate CAN as one of its key technological cores, integrating other effective therapies that are capable of regulating CPA functionality. This includes directly targeting the brain's higher centers through cognitive behavioral therapy (CBT; such as urgency exposure and reappraisal training) to reduce their catastrophic interpretation of pelvic floor signals and by strengthening the conscious connection between the cortex and muscles through pelvic floor biofeedback, thus optimizing the ascending and descending pathways of the CPA. Future approaches may integrate drug therapy, gut microbiome interventions, and even develop closed-loop regulatory systems based on wearable sensors alongside personalized digital twin models. Table 2 summarizes these multimodal intervention strategies that are applicable to different levels of the CPA.

**Table 2:** Multi-level intervention strategies and hypothesis-consistent mechanisms relevant to CPA-targeted modulation.

**CPA targeting levels**	**Core intervention technology**	**Primary target**	**Core mechanism of action**	**Reference**
Central command	rTMS (iTBS)	DLPFC	Double-blind randomized controlled trial: high-frequency rTMS applied to the DLPFC was associated with improvement in executive function and memory measures in AD patients, suggesting possible engagement of prefrontal inhibitory networks, which is consistent with (but does not establish) potential relevance to descending urinary control pathways within the CPA framework.	Wu et al., 2022
Central command	rTMS	DLPFC	Randomized crossover trial (DOC): DLPFC-rTMS improves CRS-R scores in minimally conscious patients, suggesting short-term improvements in arousal-related behavioral responsiveness, potentially via modulation of cortical–thalamic networks.	Xu et al., 2024
Spinal relay	Transcutaneous/transvertebral magnetic stimulation (tSMS/TVMS)	Thoracolumbar/sacral spinal cord	Prospective human physiological study: tSMS/TVMS was associated with changes in spinal excitability and reflex-related electrophysiological measures (e.g., evoked potentials/EMG indices), providing non-invasive physiological evidence consistent with modulation of bladder–sphincter reflex circuitry.	Kawai et al., 2024
Spinal relay	SCS (spinal cord stimulation, back column)	Dorsal column-brainstem pathway	Multicenter/clinical series: MCS/DOC patients undergoing spinal cord stimulation (SCS) reported improvement in certain consciousness indicators, suggesting potential for modulating the reticular activating/brainstem-thalamic system.	Yang et al., 2022
Peripheral input	SNM (sacral nerve modulation)	S3 sacral nerve root	Prospective multicenter study: ARTISAN-SNM demonstrates significant reduction in urinary incontinence episodes over 2 years in UUI patients, suggesting durable clinical benefit in UUI and remaining consistent with the hypothesis that sustained peripheral stimulation may modulate peripheral-to-central signaling relevant to urge/salience processing (without establishing a direct central mechanism).	Pezzella et al., 2021
Peripheral input	PTNS (percutaneous tibial nerve stimulation)	Tibial nerve → sacral spinal cord	Randomized double-blind placebo-controlled RCT: PTNS significantly reduced urge incontinence and urinary frequency, supporting a bottom-up neuromodulation approach for OAB symptoms, with effects hypothesized to involve sacral gating and/or downstream central reprocessing.	Peters et al., 2010
Integrative	Pelvic floor biofeedback (PFM-BF)	Pelvic floor muscles ↔ somatosensory cortex	Randomized controlled clinical trial: EMG-BF combined with PFMT reported additional benefits over PFMT alone in reducing objective urinary leakage and subjective symptoms, consistent with the view that cortico-muscular coordination can be behaviorally trained and may be modifiable in UI rehabilitation.	Wang et al., 2024

**Figure 3:**
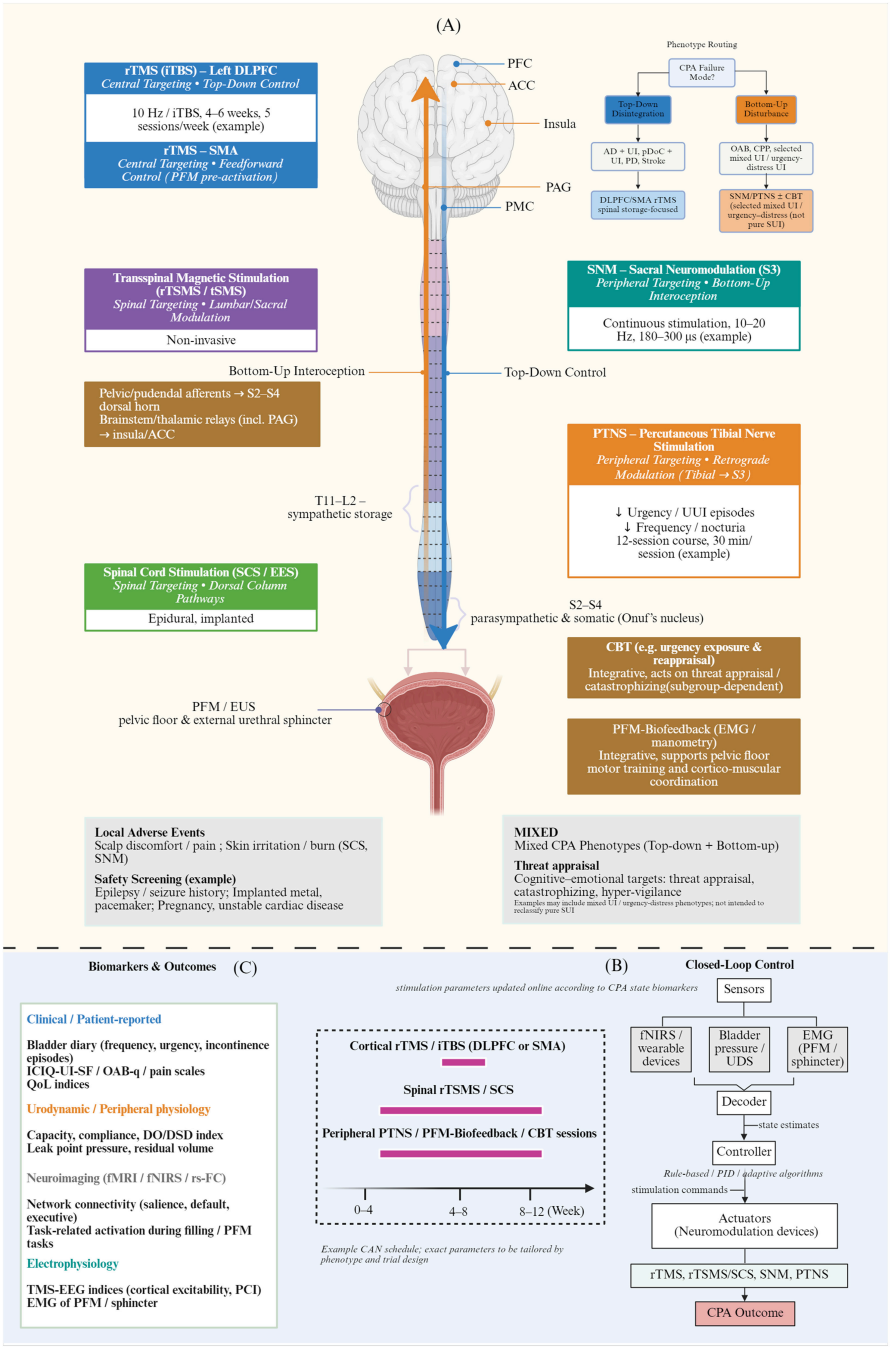
Multi-level intervention map targeting the cerebro-pelvic axis (CPA) and conceptual diagram of collaborative axis regulation (CAN). This diagram illustrates a hierarchical intervention strategy targeting different levels of the CPA and its integrated application framework. **(A)** Multi-level interventional toolkit. The vertical axis schematizes a brain–spinal–pelvic control loop. Bottom-up afferent signaling is conveyed by pelvic/pudendal pathways to the sacral dorsal horn (S2–S4), with ascending projections via relay/gating nodes (including PAG) and thalamo-cortical interoceptive–salience regions (insula/ACC; orange). Top-down control is conveyed from prefrontal networks via brainstem micturition circuitry (including PMC) to spinal autonomic/somatic segments (blue). Intervention options are positioned by level: cortical (rTMS/iTBS; DLPFC/SMA), spinal (rTSMS/SCS), and peripheral (SNM/PTNS), with CBT and pelvic-floor biofeedback as integrative adjuncts. Bottom-up relevance is restricted to phenotyped mixed UI or urgency/distress-prominent subgroups (not pure SUI). **(B)** CAN Strategy Framework: based on CPA failure modes, the upper-right phenotypic classification categorizes patients into “top-down disintegration” types [e.g., Alzheimer's disease (AD)/persistent disorders of consciousness (pDoC)] and “bottom-up disturbance”–relevant types [e.g., overactive bladder (OAB), chronic pelvic pain (CPP), and selected SUI/mixed UI phenotypes with prominent urgency/distress rather than pure SUI broadly], thereby enabling the development of personalized intervention combinations. The central timeline illustrates examples of temporal coupling between cortical, spinal cord, and peripheral stimulation when administered within the same treatment period (e.g., 0–12 weeks). The closed-loop control (Closed-Loop Control) in the lower right corner depicts a future vision where stimulation parameters are adjusted in real time via a decoder and controller using multimodal sensors such as fNIRS, urodynamics, and EMG. **(C)** Biomarkers and Outcomes: The lower left corner lists multidimensional assessment metrics, encompassing clinical symptom scales (e.g., ICIQ-UI-SF), urodynamic parameters (e.g., DO/DSD), and functional magnetic resonance imaging (fMRI) alongside electrophysiological (TMS-EEG/EMG) indicators. This schematic is hypothesis-generating and intended to guide future empirical testing. CPA, cerebro-pelvic axis; CAN, coordinated axis neuromodulation; rTMS, repetitive transcranial magnetic stimulation; iTBS, intermittent theta-burst stimulation; DLPFC, dorsolateral prefrontal cortex; SMA, supplementary motor area; PFC, prefrontal cortex; ACC, anterior cingulate cortex; PAG, periaqueductal gray; PMC, pontine micturition center; rTSMS, repetitive transspinal magnetic stimulation; SCS, spinal cord stimulation; SNM, sacral neuromodulation; PTNS, percutaneous tibial nerve stimulation; PFM, pelvic floor muscles; CBT, cognitive-behavioral therapy; PFM-BF, pelvic floor muscle biofeedback; EMG, electromyography; AD, Alzheimer's disease; pDoC, prolonged disorders of consciousness; SUI, stress urinary incontinence; OAB, overactive bladder; CPP, chronic pelvic pain; UDS, urodynamics; DO, detrusor overactivity; DSD, detrusor-sphincter dyssynergia; fMRI, functional magnetic resonance imaging; fNIRS, functional near-infrared spectroscopy; TMS-EEG, transcranial magnetic stimulation–electroencephalography.

Based on the above approach, we attempted to map interventional strategies targeting different levels of the CPA (cortical, spinal, peripheral) to corresponding clinical phenotypes of distinct failure modes (top-down disintegration vs. upward disturbance) by generating a visual multi-level intervention map. As shown in Figure 3, this map vertically organized various neuromodulation techniques along the structural hierarchy of the CPA. Horizontally, the map illustrates integrated therapeutic pathways for future clinical validation, guided by the principles of CAN for temporal coupling and closed-loop control.

### 5.3 Safety considerations and implementation challenges

CAN is intended as a conceptual scaffold to generate testable protocols; its clinical utility remains to be established. However, the combined application of multiple neuromodulation techniques introduces complex safety challenges, and their potential cumulative biological effects must be carefully evaluated. First, we need to consider alterations in cortical excitability and seizure threshold. Intense stimulation from high-frequency interventions in the cortex, spinal cord and periphery may modify cortical excitability through metaplasticity mechanisms, thereby increasing the risk of seizures in patients with stroke, TBI, and other conditions (Lu et al., 2015; Dixon et al., 2016). Second, we need to consider autonomic dysregulation. Given that CPA integrates extensively into the autonomic nervous system, it is possible that multi-point stimulation may trigger unexpected fluctuations in blood pressure, abnormal variability in heart rate, or even autonomic dysreflexia (Harkema et al., 2018). Furthermore, prolonged stimulation involving emotional centers (such as the ACC and amygdala) may warrant monitoring for neuropsychiatric adverse effects in vulnerable populations, although the available evidence is indirect and largely extrapolated from other neuromodulation settings (Boel et al., 2016).

Therefore, CAN should currently be explicitly positioned as a therapeutic paradigm in the exploratory phase, with its clinical translation strictly adhering to the ethical baseline of “do no harm.” Future clinical trials must explore rigorous safety monitoring systems that, beyond routine adverse event documentation, incorporate electroencephalogram (EEG) monitoring, real-time cardiovascular autonomic assessment, and the detailed tracking of emotional/cognitive scales. The design of future studies should employ a stepwise dose-escalation strategy with strict inclusion and exclusion criteria (excluding participants with high-risk epilepsy or unstable cardiovascular history). Until sufficient safety data has been collated from randomized controlled trials in multiple centers, the large-scale and indiscriminate use of multimodal neuromodulation combinations in highly vulnerable populations should be avoided in routine clinical practice. Early-phase studies should prioritize lower-risk cohorts and conservative dosing before extension to vulnerable populations (advanced dementia/pDoC). In frail populations, feasibility constraints (treatment time, transport, positioning, caregiver burden, and fluctuating arousal/autonomic stability) may be as limiting as physiological risk, and should be explicitly captured as outcomes in future trials.

## 6 Discussion and future outlook

Herein, we propose CPA as a hypothesis-generating control-loop mapping framework and outline “neuro-pelviology” as an interdisciplinary research agenda to organize mechanism-oriented phenotyping and translational studies across brain–pelvis interactions. To translate this vision from theory into clinical practice, future research must advance synergistically across multiple dimensions, including enhancing our understanding of mechanisms, driving technological innovation, strengthening community building and methodological standardization, and expanding applications.

### 6.1 Deepening mechanistic understanding: from macro-level circuits to micro-level analysis

Although the CPA framework provides a systematic theoretical perspective for understanding interactions between the brain and pelvic floor, we must remain mindful of current limitations. First, the specific anatomy of the human urinary center (the PMC) differs from that of classical animal models such as cats, thus necessitating caution in translational research. Second, existing non-invasive neuroimaging techniques remain limited in terms of achieving dynamic resolution for the fine structures of the brainstem and spinal cord. Finally, the optimal timing parameters for CAN therapy and its long-term efficacy still require further validation through large-scale clinical trials.

In light of these challenges, future research should first focus on addressing gaps in our current understanding of the underlying mechanisms. Although we have now mapped the macro-neural circuitry of the CPA, the micro-foundations of this axis remain largely unknown and require further systematic analysis. Basic research should explore cellular atlases for key nodes along the CPA using single-cell/spatial transcriptomics to reveal their unique molecular characteristics. Leveraging cutting-edge tools such as optogenetics/chemogenetics, viral tracing, and *in vivo* electrophysiology, future research in animal models should meticulously dissect the causal circuits connecting the higher-order networks of the brain with the pelvic autonomic center. Human research is equally important; there is an urgent need to develop and standardize multimodal synchronous recording paradigms integrating neuroimaging (fMRI/fNIRS), electrophysiology (TMS-EEG) and urodynamics/electromyography. This may enable the rationale for a reproducible biomarker system for quantifying CPA status.

### 6.2 Innovative diagnostic and therapeutic paradigms

The CPA framework provides a clear direction for innovation in clinical diagnosis and treatment. In terms of diagnostics, a promising frontier lies in exploring whether pelvic floor function parameters, such as urodynamic and EMG characteristics, can serve as early or concomitant biomarkers for the progression of CNS diseases such as AD. Therapeutically, future strategies could transcend current open-loop stimulation toward the possibility of closed-loop precision control. This is likely to require wearable sensors (such as bladder pressure/EMG patches) with advanced decoding algorithms to build a closed-loop coordinated axial neuromodulation (Closed-loop CAN) platform aiming to enable of CPA status and the automatic adjustment of stimulation parameters. Furthermore, the sequential coupling of neuromodulation with CBT and targeted medications will also be key to enhancing therapeutic efficacy. To validate these innovative solutions, there is an urgent need to design and conduct multi-center and mechanism-oriented randomized controlled trials (RCTs) targeting different CPA failure modes, such as downlink degradation-type AD + UI and uplink interference-type SUI.

### 6.3 Research community development: from initiative to consensus

To advance this interdisciplinary research agenda, a well-established academic community is required. A potential future direction would be to convene a multi-disciplinary community (e.g., an informal CPA-focused collaborative network) to enable data harmonization and cross-site studies. to develop shared governance protocols for data and sample sharing (FAIR principles), develop a potential reporting extension for CPA-focused trials (future work), ideally in alignment with existing CONSORT guidance and through broad community consensus. Simultaneously, by launching thematic issues in top-tier journals and promoting the rationale for interdisciplinary programs for talent development, we will progressively build an collaborative research ecosystem for neuro-pelviology.

### 6.4 Expanding application boundaries

The explanatory power of the CPA framework extends far beyond the few disorders highlighted in this article. The potential of CPA for broader applications remains to be explored, such as elucidating how stress and mental health issues (e.g., anxiety, PTSD) can trigger pelvic floor dysfunction through the CPA pathway. It is possible that the CPA may provide new targets for central-peripheral coordinated interventions in sexual dysfunction and postpartum rehabilitation, and offer novel monitoring and countermeasures for alterations in the autonomic nervous system in the pelvic floor in spaceflight and extreme environment medicine.

The future advancement of CPA-guided brain–pelvis translational research depends upon enhanced mechanistic research, achieving closed-loop personalized treatment, and building a robust collaborative research ecosystem. Through a clear 5-year translational roadmap, from completing the cellular atlas and task paradigm for the CPA, to initiating multi-center RCTs, and ultimately publishing clinical guidelines and establishing an open data platform, this framework holds significant promise to evolve from a conceptual model into a potential interdisciplinary translational research framework with potential for clinical translation, capable of substantially improving quality-of-life for a large number of patients.

This paper integrates neuroscience, urology and rehabilitation medicine to provide a unified analytical framework, the CPA, to enhance our understanding of the association between higher brain functionality and fundamental pelvic floor physiology. Within the CPA framework, urinary incontinence associated with AD and pDoC is reframed as a potentially informative manifestation of descending control-pathway decompensation, rather than being treated solely as an independent late-stage complication. Likewise, OAB—and a subset of SUI/mixed UI—may involve symptom-related engagement of interoceptive and executive-control networks. These CNS effects are framed as testable associations, not proven causal remodeling. Building upon this foundation, the neuro-pelviology research agenda that we suggest represents a crucial pathway for translating this theoretical understanding into clinical practice. This concept uses the control loop as a unit of analysis and failure modes as a shared analytic vocabulary for cross-condition phenotyping, aiming to achieve holistic restoration of entire axis functionality through multi-level interventional strategies such as CAN. To mature this research agenda into a robust translational program, it is necessary to explore common data standards, form interdisciplinary alliances, and conduct mechanism-oriented clinical trials. This will enable holistic restoration of CPA function, ultimately improving quality-of-life for patients.

## Data Availability

The original contributions presented in the study are included in the article/supplementary material, further inquiries can be directed to the corresponding authors.

## Abbreviations

5-HT, 5-hydroxytryptamine (serotonin); 7T-fMRI, 7-tesla functional magnetic resonance imaging; ACC, anterior cingulate cortex; ACh, acetylcholine; AD, Alzheimer's disease; AD+UI, Alzheimer's disease plus urinary incontinence; ALE, activation likelihood estimation (meta-analytic method for fMRI); ARTISAN-SNM, ARTISAN-SNM clinical study of a rechargeable sacral neuromodulation system for urinary urgency incontinence; BPS, bladder pain syndrome; CBT, cognitive-behavioral therapy; CAN, coordinated axis neuromodulation; CNS, central nervous system; CONSORT-CPA, consolidated standards of reporting trials – cerebro-pelvic axis extension; CPA, cerebro-pelvic axis; CPA-Net, Cerebro-Pelvic Axis Research Alliance Network; CPP, chronic pelvic pain; CRS-R, Coma Recovery Scale–Revised; DA, dopamine; DLPFC, dorsolateral prefrontal cortex; DOC, disorders of consciousness; DO, detrusor overactivity; DSD, detrusor–sphincter dyssynergia; EES, epidural electrical stimulation; EEG, electroencephalography; EMG, electromyography; EMG-BF, electromyography-based biofeedback; FAIR, findable, accessible, interoperable, reusable (data principles); fMRI, functional magnetic resonance imaging; fNIRS, functional near-infrared spectroscopy; IC, interstitial cystitis; ICIQ-UI-SF, International Consultation on Incontinence Questionnaire – Urinary Incontinence Short Form; ICI-RS, International Consultation on Incontinence – Research Society; iTBS, intermittent theta-burst stimulation; LURN, Symptoms of Lower Urinary Tract Dysfunction Research Network; LUT, Lower urinary tract; MAPP, Multidisciplinary Approach to the Study of Chronic Pelvic Pain (Research Network); MCS, minimally conscious state; MIBG, meta-iodobenzylguanidine; NE, norepinephrine; NDO, neurogenic detrusor overactivity; OAB, overactive bladder; PAG, Periaqueductal gray; PAS, paired associative stimulation; PD, Parkinson's disease; PFC, prefrontal cortex; PFM, pelvic floor muscles; PFM-BF, pelvic floor muscle biofeedback; PFMT, pelvic floor muscle training; PMC, pontine micturition center; PTNS, percutaneous tibial nerve stimulation; PTSD, post-traumatic stress disorder; pDoC/pDOC, prolonged (persistent) disorders of consciousness; RCT, randomized controlled trial; rFMS, repetitive functional magnetic stimulation; rTMS, repetitive transcranial magnetic stimulation; rTSMS, repetitive transspinal magnetic stimulation; rs-fMRI, resting-state functional magnetic resonance imaging; SCS, Spinal cord stimulation; SMA, supplementary motor area; SNM, sacral neuromodulation (sacral nerve modulation); SUI, stress urinary incontinence; TBI, traumatic brain injury; TMS-EEG, transcranial magnetic stimulation–electroencephalography; TMSCS, transcutaneous magnetic spinal cord stimulation; tSMS, transcutaneous magnetic stimulation; TVMS, transvertebral magnetic stimulation; UDS, urodynamic studies; UI, urinary incontinence; UUI, urgency urinary incontinence.

## Glossary

Cerebro-pelvic axis (CPA): A hypothesis-generating control-architecture mapping/phenotyping framework linking higher brain networks to pelvic floor and lower urinary tract control via descending executive control and ascending interoceptive feedback loops.

Neuro-pelviology: An interdisciplinary research agenda integrating neuroscience, urology/pelvic-floor medicine, and rehabilitation to study CPA-relevant control-loop phenotypes, measurable coupling readouts, and mechanism-oriented intervention designs.

Descending control pathway (top-down control): Supraspinal executive and contextual control over brainstem/spinal micturition circuits enabling continence and socially appropriate voiding decisions.

Ascending interoceptive pathway (bottom-up signaling): Afferent pelvic signals (e.g., bladder filling/urgency, pain, leakage awareness) transmitted to interoceptive hubs (insula/ACC) shaping subjective experience and behavior.

Top-down disintegration: CPA failure mode driven primarily by central network impairment (e.g., AD, pDoC), leading to loss of voluntary inhibition and reflex-dominant voiding.

Bottom-up disturbance: A CPA failure-mode category primarily involving persistent or recurrent abnormal pelvic afferent/interoceptive load (most directly supported in conditions such as OAB and CPP), with possible subgroup-dependent applicability to mixed UI or SUI with prominent urgency/distress. It is hypothesized to be associated with heightened vigilance and, in some cases, central network adaptation.

Coordinated axis neuromodulation (CAN): A proposed multi-level neuromodulation paradigm that temporally and mechanistically couples cortical, spinal, and peripheral interventions to restore CPA function.

CPA circuitopathy: A conceptual term describing maladaptive CPA-level dysfunction arising from aberrant ascending signaling and consequent central plasticity changes.
